# Personality, Coping and Developmental Conditions in Female Adolescents and Young Adults with Type 1 Diabetes: Influence on Metabolic Control and Quality of Life

**DOI:** 10.3389/fpsyt.2021.809015

**Published:** 2022-03-10

**Authors:** Gudrun Wagner, Michael Zeiler, Andreas Karwautz, Andrea Schneider, Birgit Rami-Merhar, Gabriele Berger

**Affiliations:** ^1^Eating Disorders Unit at the Department of Child and Adolescent Psychiatry, Medical University of Vienna, Vienna, Austria; ^2^Department of Pediatrics and Adolescent Medicine, Medical University of Vienna, Vienna, Austria; ^3^Pediatric Diabetes Outpatient Clinic, Health Care Centre Vienna Floridsdorf, Vienna, Austria

**Keywords:** adolescents, metabolic control, personality, autonomy, developmental conditions

## Abstract

**Objective:**

To assess personality factors, coping, developmental conditions and quality of life in female adolescents and young adults with type 1 diabetes (T1D) and high vs.low HbA1c.

**Methods:**

Patients were approached at the Department for Pediatrics, Medical University of Vienna; *n* = 129 female adolescents (10 to 23 years, mean age 15.21 ± 2.91) with type 1 diabetes were included. HIGH-A1c was defined as HbA1c > 7.5%, LOW-A1c as HbA1c ≤ 7.5% and compared to a sample of 56 age-matched female healthy controls. Self-rating questionnaires were used to assess psychosocial factors: *Children's Depression Inventory* (CDI); *Junior Temperament and Character Inventory* (J-TCI); *Eating Disorders Inventory-2* (EDI-2); *KIDCOPE; Subjective Family Image Test* (SFIT) and *Inventory of Life Quality in Children and Adolescents*(ILC).

**Results:**

T1D patients with HIGH-A1c were younger at the age of diabetes onset, had a longer diabetes duration, a higher maximum BMI, higher depression score, and higher frequency of diabetic ketoacidosis in the last year. They showed significantly higher levels of fatigue, lower levels of taking responsibility, lower ability to set goals and lower self-acceptance, as well as higher levels of ineffectiveness, lower levels of emotional attachment within the family, in particular with the fathers, and used negative coping strategies more often compared to patients with LOW-A1c. Furthermore, they reported significantly higher burden of illness and lower quality of life.

**Conclusions:**

Disadvantageous personality and coping styles as well as developmental conditions should be addressed in the treatment of female adolescents with T1D with management problems.

## Introduction

Over the past two decades, the use of more intensive therapy regimes such as basal-bolus or continuous subcutaneous insulin infusion (CSII) has increased (1). Improvement in metabolic control over time was observed in several diabetes cohort registries (1–6), but the targeted HbA1c levels were not achieved in the majority of studies, with mean HbA1c levels ranging from 7.6 to 8.9% (6, 7), and a deterioration of HbA1c in adolescent patients (7) was described in all surveys mentioned above.

While of all young patients <18 years 37% reach a HbA1c of <7.5% (3), in adolescents between 13 and 20 years, only 21% reach HbA1c levels of <7.5% (8). In particular, adolescent girls with longer diabetes duration show the highest HbA1c (2) and are at risk of diabetic ketoacidosis (7, 9). Therefore, adolescence and young adulthood is recognized as a time period with high risk of deterioration of metabolic control (10, 11).

It has been stated that important determinants of metabolic control are non-medical, but rather psychosocially based (12–16). Children and adolescents with chronic poor metabolic control are more likely to have psychosocial problems compared to those with good metabolic control (17). Moreover, the diabetes regimen seems to be only as good as the ability and motivation of the child and family to manage it, reflecting the role of self-management abilities and family factors for metabolic control (18). Previous studies have shown that socioeconomic status, family stress, family climate (19), and family conflicts (20) have an influence on metabolic control, as well as the degree of parental involvement in diabetes care (21, 22). To date there are no studies on the impact of general familial developmental conditions and family relations.

Female adolescents have been shown to be at higher risk of developing internalizing psychiatric disorders, such as depression (23, 24), which in turn increases the risk for suboptimal development including low illness functioning and low self-esteem (25). Examination of personality factors revealed strong associations between conscientiousness, self-efficacy, self-discipline and cautiousness with good diabetes adherence, whereas high levels of neuroticism, anger, depression and impulsiveness were related to low adherence in an adolescent population (26). Furthermore, it has been recommended to analyze personality traits and self-esteem in different models for adjustment to chronic illness as they represent core features of a person's self-concept. Personality traits and self-esteem are presumed to be important for glycemic control, treatment adherence, quality of life and coping in T1D (27). Recent research has demonstrated lower self-esteem, less emotional stability and lower treatment adherence in young adult women with T1D as compared to young adult men with T1D, with depressive symptoms functioning as a mediator (27–30). Small sample sizes and the absence of objective measures for glycemic control (HbA1c) have limited previous research. According to the transactional stress and coping model (31), adjusting to chronic illness is achieved by a complex interplay of demographic and clinical parameters, as well as coping skills and self-perception. A weak sense of oneself may put young people at risk for maladjustment.

Studies show that a good glycemic control is associated with positive quality of life (QoL). QoL is a multidimensional construct including physical, emotional and psychosocial well-being of an individual and can be regarded as important variable to evaluate treatment outcome (32).

In summary, individual and family related psychosocial factors influence therapy adherence and subsequently metabolic control and quality of life in adolescents—especially in females—reflecting a complex interplay of personality traits, coping strategies, family interactions and depressive symptoms.

Therefore, the aim of our study was to assess personality characteristics, coping strategies, individual autonomy and emotional connectedness within the family, depression and quality of life in female adolescents with T1D with low vs. high HbA1c compared to healthy controls without a chronic condition in order to address the association of these factors with metabolic control in female adolescents.

## Methods

### Procedure

Female patients with T1D within the age range of 10 to 23 years and diabetes duration of ≥1 year were approached at the University Clinic for Pediatrics and Adolescents, Department for Endocrinology at the Medical University of Vienna. The study protocol was approved by the Ethics Committee of the Medical University of Vienna (272/2003), and written consent was obtained from participants as well as from parents in case of minors. Exclusion criteria were a second chronic condition as i.e., celiac disease, as well as verbal and intellectual disability. An age-matched female control group without a chronic condition was recruited from the general population.

### Measures

#### Glycemic Control

HbA1c was used as an objective measure of metabolic control. HbA1c values were assessed during a routine diabetes outpatient visit (DCA Vantage, Siemens). Individuals with values > 7.5% were categorized into the HIGH-A1c group and individuals with values ≤ 7.5% were categorized into the LOW-A1c group.

#### Depression

The Children's Depression Inventory (CDI) (33) measures severity of depression in children and adolescents. It comprises 27 items, including statements of typical symptoms of depression, but also side effects and consequences. Findings suggest a total score of ≥ 18 differentiating between patients with clinically relevant depression and healthy individuals. Internal consistency as obtained in a sample of psychopathological children (α = 0.88) and healthy controls (α = 0.85), respectively are good. Split-half reliability of *r* = 0.91 and *r* = 0.84 in the clinical and healthy group, respectively is high.

#### Eating Disorders Psychpathology

The Eating Disorders Inventory-2 (EDI-2) (34) is a self-report questionnaire, originally designed for the assessment of attitudinal and behavioral dimensions relevant to eating disorders but comprises subscales relevant for more general and potential problematic personality traits in adolescence. Besides eating disorders subscales such as “drive for thinness,” “body dissatisfaction” and “bulimia,” the subdomains “ineffectiveness,” “perfectionism,” “interpersonal distrust,” “interoceptive awareness,” “maturity fears,” “asceticism,” “impulse regulation” and “social insecurity” are assessed. In total, 11 subscales are derived from 91 items presented in a six-point, forced choice Likert scale. The German version shows good validity and reliability. Cronbach's α is .98 for the total score and range from 0.70 to 0.94 for the subscales. Test-retest reliability within 4 months is satisfactory for the total score with *r* = 0.78. For all other subscales test-retest reliability coefficients range from *r* = 0.42 to *r* = 0.84, except asceticism *r* = 0.12 (35).

#### Personality

To assess relevant dimensions of personality factors we administered the dimension “harm avoidance” and “self-directedness” of the Junior Temperament and Character Inventory (J-TCI) (36). Harm avoidance belongs to the temperament dimensions, considered as automatic emotional reaction in the psycho-biological personality model of Cloninger (37). Harm avoidance (HA) consists of the subscales future concerns (HA1), fear of uncertainty (HA2), timidity (HA3), fatigue (HA4). Self-directedness (SD) is part of the character scales describing differences in the self-concept and includes attitudes, values and aims as well as the capacity of dealing with the environment. Self-directedness covers the subscales responsibility (SD1), the ability to set goals (originally named purposefulness, SD2), the ability to follow goals (originally named resourcefulness, SD3), and self-acceptance (SD4). The J-TCI is a self-rating questionnaire, with 5-point Likert scaled item rating, with higher values indicating higher levels of agreement. Internal consistency of the global HA and SD scales are α = 0.83 and α = 0.84; the retest reliability is *r* = 0.76 for both scales. We have chosen HA and SD because there is evidence for an association between these subscales for axis-I mental health problems (36, 38).

#### Coping Strategies

The KIDCOPE (39) is a self-report measure of coping strategies with disease-related and everyday problems in children and adolescents based on the Lazarus and Folkman theory of stress and coping (40). We assessed “wishful thinking” as one example of avoidance coping strategies as well as “self- blame” and “blaming others” as examples of negative coping strategies as these strategies have been demonstrated to be applied more often in diabetes patients with adherence problems (38). Patients are instructed to imagine difficult situations concerning their diabetes management and asked to indicate how often they apply each of these coping strategies (never, sometimes, often, most of the time). For the purpose of this study, we dichotomized this variable into “coping strategy never used” and “coping strategy used” (sometime to most of the time).

The adolescent version of the KIDCOPE was used for the entire sample as it turned out comprehensible also for older children aged between 10 and 12 years. Coping is conceptualized as a process measure and not a stable personality trait resulting in rather limited stability with low retest reliabilities after 10 weeks (*r* = 0.15 to 0.43).

#### Family Relations

The self-rating instrument Subjective Family Image Test (SFIT) (41) was used to assess subjective perceptions of relationships within the family (father-mother-child triad) from the perspective of the adolescent. It has two subscales, “individual autonomy” (IA) and “emotional connectedness” (EC). Six pairs of adjectives are rated on a 7-point Likert scale (−3 to +3). The IA scale is derived from three pairs of adjectives (confident—anxious, independent—dependent, decisive—indecisive). The EC scale is also derived from three adjective pairs (understanding—intolerant, warm-hearted—cool, interested—disinterested). The following scores can be calculated:

1. Individual relationships: Patients' perceptions of their mutual relationship toward father and mother and the relationship of the parents to each other are assessed in IA and EC.
2. Each perceived relationship has a minimum value of −9 and maximum value of +9, since each scale (EC or IA) is derived from three adjective pairs with a range from −3 to +3.
3. Family sums: All individual relationships are summed up to two family sum scores, leading to “Family sum autonomy” and “family sum emotional connectedness,” both in the value range of −54 to +54.
4. Developmental conditions correspond mathematically to the sum of family sum autonomy + family sum emotional connectedness ranging from minimum −108 to maximum of +108. High values are considered to be optimal development conditions, including high individual autonomy and high emotional connectedness. High values are regarded as positive family resources. Likewise low developmental conditions including low autonomy and low emotional connectedness represent unfavorable developmental conditions and negative familial factors.
5. Cohesion is calculated as difference “Family sum EC” minus “Family sum IA” with values ranging from −108 to +108. Higher values represent higher family cohesion (high EC and low IA) without the possibility for the development of individual autonomy (high attachment with inhibiting development). Lower values (high autonomy with low EC) represent high autonomy with inhibiting development.

Cronbach's α for the IA scale is 0.61, and 0.81 for the EC scale; parallel-test reliability ranged from *r* = 0.61 to *r* = 0.80, test-retest reliability after 2 weeks from *r* = 0.66 to *r* = 0.82, and after 11 months from *r* = 0.55 to *r* = 0.69 (38, 42).

#### Quality of Life

The Inventory of Life Quality in Children and Adolescents (ILC) (43) measures subjective well-being and subjective satisfaction with different areas of physical and psychological conditions as well as with social contexts in life. Subjective well-being or satisfaction is self-reported by the patients. Nine different areas are assessed: school, family, peers, interests and activities, physical health, psychological health and overall QoL judgement. Two items addressing the burden of disease and therapy associated to diabetes have been added. The ILC is a 9 item self-rating questionnaire; response categories are 5-point Likert-scaled. Internal consistencies (Cronbach's alpha) range between 0.55 and 0.76, re-test reliabilities for the total score range between *r* = 0.60 and *r* = 0.80. The ILC is known as an economically applicable instrument for the assessment of QoL in children and adolescents. It is a widely used instrument in many European countries for evaluation and quality assurance of treatments of children and adolescents with chronic physical and mental conditions.

### Statistics

The statistical analyses were performed with IBM SPSS Statistics 25.0. First, sociodemographic and diabetes-specific characteristics were compared between the groups (patients with high vs. low HbA1c and. healthy controls) using ANOVA or *t*-tests for continuous variables and χ^2^-tests for categorical variables. Next, we conducted a series of (univariate) general linear models to analyze differences in personality scores (J-TCI, EDI-2), coping strategies (KIDCOPE), subjective family image scores (SFIT) and QOL scores between the three groups. For questionnaire subscales, we used Bonferroni-adjusted significant levels to account for multiple comparisons. As the individual sum comparisons in the SFIT were analyzed on an exploratory level only, no adjustment of the significant level was applied here. In the case of statistically significant overall group differences, Tukey *post-hoc* tests were performed to explore individual group differences. We used χ^2^-tests to analyze differences in the applied coping strategies (yes vs. no) between the patients with good vs. suboptimal metabolic control. Finally, we applied logistic regression analyses in patients with T1D with low HbA1c (compared to high HbA1c) as dependent variables. In a first step, we performed a series of univariate logistic regressions using sociodemographic, diabetes-specific characteristics, as well as personality, coping, family relation variables as independent variables (see Supplementary Table S1 in the electronic Supplementary Material). All independent variables with *p* <0.200 in the univariate analyses were further considered as predictors in a multivariate logistic regression analysis. We used a stepwise procedure with three blocks of predictors (1st block: socio-demographic and diabetes-specific variables: 2nd block: personality-associated variables; 3rd family-relation variables). Of note, in a multivariate regression model, independent variables are controlled for all other variables included in the model which is important with regard to group differences (e.g., depression scores) presented in Table 1.

**Table 1:** Sociodemographic and diabetes specific parameters.

	**T1D patients with HIGH-A1c^†^ (*N* = 76) a**	**T1D patients with LOW-A1cc^†^ (*N* = 53) b**	**Female Healthy controls (*N* = 56) c**	**Test statistic, *p***	***Post-hoc* comparisons (significant differences)**
	**Mean (SD)**	**Mean (SD)**	**Mean (SD)**		
Age (years)	15.21 (2.91)	14.40 (2.93)	14.66 (2.77)	*F* = 1.321, *p* = .270	
**Age at diabetes diagnoses (years)**	**8.35 (3.64)**	**9.86 (3.73)**	**-**	***t*** **=** **2.262**, ***p*** **=** **.025**	**a** **<** **b**
**Diabetes duration (years)**	**6.86 (4.05)**	**4.57 (3.29)**	**–**	***t*** ** = −3.488**, ***p*** **=** **.001**	**a** **>** **b**
**HbA1c**	**9.19 (1.41)**	**6.87 (0.49)**	**–**	***t*** ** = −11.489**, ***p*** **<** **.001**	**a** **>** **b**
**BMI current**	**21.95 (3.99)**	**20.62 (3.24)**	**19.77 (3.14)**	***F*** **=** **6.195**, ***p*** **=** **.003**	**a** **>** **c**
**BMI maximum**	**23.56 (4.17)**	**21.86 (3.60)**	**20.25 (3.39)**	***F*** **=** **11.260**, ***p*** **<** **.001**	**a** **>** **c**
**Depression Total Score (CDI)**	**12.55 (7.15)**	**7.79 (4.92)**	**8.53 (4.15)**	***F*** **=** **12.144**, ***p*** **<** **.001**	**a** **>** **b, a** **>** **c**
	**%**	**%**	**%**	**χ^2^, p**	
Parents living in partnership	87.7	86.0	86.5	*χ^2^* = 0.079, *p* = 0.961	
Treatment modalities					
Conventional insulin treatment	6.6	7.5	–	*χ^2^* =0.362, *p* = 0.834	
Basal bolus insulin therapy (FIT)	81.6	77.4	–		
Continuous subcutaneous insulin infusion	11.8	15.1	–		
Diabetes in the family (% yes)	31.1	30.0	–	*χ^2^* = 0.016, *p* = 0.898	
Hypos last year (% yes)	34.2	22.6	–	*χ^2^* = 2.011, *p* = 0.156	
**Ketoacidosis last year (% yes)**	**18.4**	**5.7**	**–**	***χ^2^* = 4.444, *p* = 0.035**	
Microalbuminuria (% yes)	9.9	9.8	-	*χ^2^* <0.001 *p* = 0.992	

† *HIGH-A1c was defined as HbA1c higher than 7.5%, LOW-A1c as HbA1c ≤ 7.5%, Lines in bold represent statistically significant group differences*.

## Results

### Participants

A total of 129 female participants with T1D and 56 healthy female controls were included in this study. We classified 76 (59%) patients with an HbA1c > 7.5% (58,2 mmol/l) as patients with “HIGH-A1c” and 53 (41%) patients with an HbA1c ≤ 7.5% (58,2 mmol/l) as patients with “LOW-A1c.” Groups did not differ with regard to insulin therapy [χ^2^(1) = 0.362; *p* = 0.834], to mean age, marital status of the parents, diabetes in other family members, frequency of hypoglycemia and microalbuminuria (see Table 1). Patients with HIGH-A1c were significantly younger at the age of diagnosis, had a longer diabetes duration and more frequent episodes of diabetic ketoacidosis associated with hospital admissions during the preceding year than patients with LOW-A1c (see Table 1). Current Body Mass Index (BMI) and maximum BMI were higher in patients with HIGH-A1c, compared to healthy controls (see Table 1).

### Depression

Depression scores were significantly higher in patients with HIGH-A1c compared to patients with LOW-A1c and healthy controls (see Table 1).

### Eating Disorders Psychopathology

Furthermore, patients with HIGH-A1c had a tendency toward higher drive for thinness, body dissatisfaction, and ascetism compared to patients with LOW-A1c; however, these differences were not statistically significant when considering Bonferroni-corrected significance levels. The groups did not differ with regard to other personality domains such as perfectionism, maturity fears, impulse regulation or social insecurity (see Table 2). The subscale ineffectiveness remained significant after Bonferroni corrections with higher values in the HIGH-A1c group compared to the LOW-A1c group and healthy controls.

**Table 2:** Eating disorders psychopathology and personality in T1D patients with HIGH-A1c and LOW-A1c compared with healthy controls.

	**T1D patients with HIGH-A1c^†^ (*N* = 76) a**	**T1D patients with LOW-A1c^†^ (*N* = 53) b**	**Healthy controls (*N* = 56) c**	**Test statistic, *p***	***Post-hoc* comparisons (significant differences)**
	**Mean (SD)**	**Mean (SD)**	**Mean (SD)**		
**EDI-2 scales**					
Total score	41.33 (24.50)	33.04 (22.72)	29.08 (15.79)	*F = 5.299, p = .006*	a > c
Drive for thinness^§^	4.16 (4.37)	4.39 (5.49)	2.04 (2.97)	*F = 4.960, p = .008*	a > c, b > c
Bulimia^§^	0.53 (1.44)	0.54 (1.61)	0.13 (0.48)	*F* = 1.866, *p* = .158	
Body dissatisfaction^§^	9.22 (7.49)	6.71 (7.83)	5.23 (5.40)	*F = 5.377, p = .005*	a > c
**Ineffectiveness** ^**§**^	**3.73 (4.70)**	**2.13 (2.44)**	**1.67 (2.11)**	***F*** **=** **6.319**, ***p*** **=** **.002**	**a** **>** **b, a** **>** **c**
Perfectionism^§^	3.35 (3.04)	3.06 (2.99)	3.50 (2.96)	*F* = 0.298, *p* = .743	
Interpersonal distrust^§^	2.83 (3.13)	2.51 (3.44)	2.17 (2.43)	*F* = 0.749, *p* = .474	
Interoceptive awareness^§^	2.51 (2.83)	1.77 (2.59)	2.26 (2.88)	*F* = 1.094, *p* = .337	
Maturity fears^§^	5.77 (4.20)	5.42 (3.18)	5.82 (4.20)	*F* = 0.165, *p* = .848	
Asceticism^§^	2.15 (2.13)	1.45 (1.54)	1.46 (1.41)	*F = 3.347, p = .037*	none sign.
Impulse regulation^§^	2.38 (3.28)	1.75 (2.64)	1.80 (2.64)	*F* = 0.990, *p* = .374	
Social insecurity^§^	4.20 (3.47)	3.33 (3.41)	2.84 (3.45)	*F* = 2.561, *p* = .080	
**J-TCI scales**					
Harm avoidance (total)	22.56 (9.79)	21.46 (9.98)	21.45 (6.57)	*F* = 0.331, *p* = .719	
- Future concerns^‡^	6.19 (3.08)	5.88 (2.90)	6.04 (2.78)	*F* = 0.163, *p* = .849	
- Fear of uncertainty^‡^	5.37 (3.58)	4.87 (3.40)	5.65 (2.41)	*F* = 0.797, *p* = .452	
- Timidity^‡^	5.48 (3.03)	5.65 (2.97)	5.40 (2.22)	*F* = 0.116, *p* = .891	
- Fatique^‡^	5.52 (2.21)	5.06 (2.74)	4.36 (2.16)	*F = 3.814, p = .024*	a > c
**Self-directedness (total)**	**39.81 (9.85)**	**45.33 (9.42)**	**42.27 (7.78)**	***F*** **=** **5.586**, ***p*** **=** **.004**	**a** **<** **b**
- **Responsibility**^**‡**^	**11.27 (2.91)**	**12.88 (2.69)**	**11.27 (2.51)**	***F*** **=** **6.479**, ***p*** **=** **.002**	**a** **<** **b, c** **<** **b**
- **Purposefulness**^**‡**^	**8.27 (2.70)**	**9.75 (2.27)**	**8.82 (2.36)**	***F*** **=** **5.504**, ***p*** **=** **.005**	**a** **<** **b**
- Resourcefulness^‡^	7.85 (2.53)	8.15 (2.67)	7.49 (2.07)	*F* = 0.989, *p* = .374	
- **Self-acceptance**^**‡**^	**12.43 (4.41)**	**14.54 (4.18)**	**14.69 (3.32)**	***F*** **=** **6.495**, ***p*** **=** **.002**	**a** **<** **b, a** **<** **c**

† *HIGH-A1c was defined as HbA1c higher than 7.5%, LOW-A1c as HbA1c ≤ 7.5%*.

§ p-value of these subscales compared with Bonferroni-adjusted significant level of 0.004.

† *p-value of these subscales compared with Bonferroni-adjusted significant level of .0125*.

*EDI-2, Eating Disorder Inventory-2; J-TCI, Junior Temperament and Character Inventory*.

*Lines in bold represent statistically significant group differences*.

### Personality

Patients with HIGH-A1c showed significantly lower responsibility, less ability to set goals (purposefulness), and lower self-acceptance as well as higher ineffectiveness than patients with LOW-A1c and with regard to self-acceptance and ineffectiveness also significantly lower values compared to healthy controls (see Table 2).

### Coping

Patients with HIGH-A1c apply “blaming others” in stressful diabetes management situations more often compared to patients with LOW-A1c [45.3 vs. 26.9%; χ^2^(1) = 4.427; *p* = 0.035], whereas “self-criticism” and “wishful thinking” were applied equally in both groups [61.3 vs. 55.8%; χ^2^(1) = 0.393; *p* = 0.531 and 82.4 vs. 82.7%; χ^2^(1) = 0.001; *p* = 0.970].

### Family Relations

Emotional connectedness within the whole family system was weaker in patients with HIGH-A1c compared to patients with LOW-A1c and healthy controls, whereas no differences in individual autonomy were found neither in the whole family system, nor on the individual level.

Differences between groups were found with respect to developmental conditions and family cohesion with deteriorated developmental conditions and lower family cohesion in patients with HIGH-A1c (see Table 3).

**Table 3:** Subjective family image in female T1D patients with HIGH-A1c and LOW-A1c compared with healthy controls.

	**T1D patients with HIGH-A1c (*N* = 76) a**	**T1D patients with LOW-A1c (*N* = 53) b**	**Female Healthy controls (*N* = 56) c**	**Test statistic, *p***	***Post-hoc* comparisons (significant differences)**
	**Mean (SD)**	**Mean (SD)**	**Mean (SD)**		
**Family sums**					
Individual autonomy	36.23 (12.55)	39.78 (11.38)	39.27 (13.93)	*F* = 1.288, *p* = .279	
**Emotional connectedness**	**30.50 (17.06)**	**38.64 (12.29)**	**38.19 (15.73)**	***F*** **=** **5.036**, ***p*** **=** **.008**	**a** **<** **b, a** **<** **c**
**Developmental conditions**	**66.73 (26.76)**	**78.85 (21.84)**	**77.46 (28.07)**	***F*** **=** **3.698**, ***p*** **=** **.027**	**none sign**.
**Cohesion**	**−5.73 (13.46)**	**−0.71 (8.70)**	**−1.08 (9.77)**	***F*** **=** **3.559**, ***p*** **=** **.031**	**none sign**.
**Individual sum comparisons**					
Child → Mother IA	5.29 (3.39)	6.34 (3.07)	6.55 (2.77)	*F* = 2.856, *p* = .060	
Mother → Child IA	6.62 (2.42)	6.79 (2.17)	6.43 (3.87)	*F* = 0.189, *p* = .828	
Child → Mother EC	5.46 (3.69)	6.64 (2.45)	6.38 (2.97)	*F* = 2.250, *p* = .109	
Mother → Child EC	6.78 (2.96)	7.55 (1.95)	7.33 (2.98)	*F* = 1.204, *p* = .303	
Child → Father IA	5.15 (3.44)	5.86 (2.71)	5.98 (2.81)	*F* = 1.267, *p* = .285	
Father → Child IA	6.42 (3.02)	7.00 (2.41)	6.92 (2.75)	*F* = 0.726, *p* = .486	
***Child** ** → Father EC***	***3.85 (4.08)***	***6.07 (3.06)***	***5.11 (3.79)***	***F** **=** **4.740, p** **=** **.010***	***a** **<** **b***
***Father** ** → Child EC***	***4.14 (4.65)***	***6.43 (3.15)***	***5.79 (3.89)***	***F** **=** **4.739, p** **=** **.010***	***a** **<** **b***
Mother → Father IA	6.30 (2.94)	6.83 (2.82)	6.51 (3.32)	*F* = 0.390, *p* = .678	
Father → Mother IA	6.41 (2.88)	6.64 (2.86)	6.58 (3.05)	*F* = 0.096, *p* = .908	
Mother → Father EC	5.35 (4.27)	6.00 (3.46)	6.92 (3.03)	*F* = 2.685, *p* = .071	
Father → Mother EC	4.89 (4.79)	5.71 (3.77)	6.11 (4.09)	*F* = 1.237, *p* = .293	

*IA, Individual autonomy; EC, Emotional connectedness; Lines in bold represent statistically significant group differences*.

Regarding individual relations, we did not find differences in the mother-father-child triad, with the exception of emotional attachment in the bi-directional child and father relation, with emotional attachment being weaker in patients with HIGH-A1c compared to patients with LOW-A1c.

### Quality of Life

Overall quality of life, physical and psychological health have been rated as significantly lower in patients with HIGH-A1c compared to patients with LOW-A1c and healthy controls, whereas patients with LOW-A1c and healthy controls did not differ within these domains. In the school, family, peer group as well as leisure and activity domains, patients with HIGH-A1c, patients with LOW-A1c and healthy controls did not differ significantly. With regard to perceived burden of diabetes and diabetes treatment and management, patients with HIGH-A1c experienced significantly higher burden in both domains compared to patients with LOW-A1c (see Table 4).

**Table 4:** Quality of life in T1D patients with HIGH-A1c and LOW-A1c compared with healthy controls.

	**T1D patients with HIGH-A1c (*N* = 76) a**	**T1D patients with LOW-A1c (*N* = 53) b**	**Healthy controls (*N* = 56) c**	**Test statistic, *p***	**Post-hoc comparisons (significant differences)**
	**Mean (SD)**	**Mean (SD)**	**Mean (SD)**		
**Overall Quality of Life^†^**	**20.68 (4.00)**	**23.08 (3.45)**	**22.82 (2.96)**	***F*** **=** **8.866**, ***p*** **<** **.001**	**a** **<** **b, a** **<** **c**
School^†^^§^	4.04 (0.92)	4.25 (0.76)	3.85(0.91)	*F* = 2.725, *p* = .068	
Family^†^^§^	4.17 (0.82)	4.58 (0.70)	4.50 (0.86)	*F* = 4.668, *p* = .011	a < b
Leisure^†^^§^	4.44 (0.84)	4.58 (0.61)	4.69 (0.54)	*F* = 2.005, *p* = .138	
Interests & Activities^†^^§^	4.14 (0.83)	4.27 (0.87)	4.22 (0.79)	*F* = 0.392, *p* = .676	
**Physical health^†^^§^**	**3.60 (0.91)**	**4.10 (0.75)**	**4.22 (0.66)**	***F*** **=** **11.057**, ***p*** **<** **.001**	**a** **<** **b, a** **<** **c**
**Psychological health^†^^§^**	**3.49 (1.01)**	**3.96 (0.86)**	**4.02 (0.73)**	***F*** **=** **6.996**, ***p*** **=** **.001**	**a** **<** **b, a** **<** **c**
**Disease-associated burden^‡^**	2.61 (1.08)	2.08 (0.91)	-x-	*t* = 2.866, *p* = .005	a > b
**Therapy-associated burden^‡^**	2.79 (1.11)	2.18 (0.99)	-x-	*t* = 3.156, *p* = .002	a > b

† *High values indicate high quality of life*.

‡ *High values indicate high burden*.

§ *p-value of these subscales compared with Bonferroni-adjusted significant level of 0.008*.

*Lines in bold represent statistically significant group differences*.

### Regression Model for Suboptimal Metabolic Control

In the multivariate hierarchical logistic regression model, the first block of independent variables (sociodemographic and diabetes-specific variables) explained about 21% of the difference in patients with HIGH-A1c vs. LOW-A1c [χ^2^(2) = 15.643; *p* <0.001: Nagelkerke *R*^2^ = 0.206]. By adding personality-associated variables to the model in the next block, the model fit significantly improved [Nagelkerke *R*^2^ = 44%; Δ model fit: χ^2^(7) = 21.384; *p* = 0.003]. Adding the third block of independent variables (family relation), no further significant increase in the model fit [Nagelkerke R^2^: 47%; Δ model fit: χ^2^(2) = 3.118; *p* = 0.210] occurred. In the final model (Table 5), only the duration of illness was significantly associated with the HbA1c status with longer illness duration associated with the risk for HIGH-A1c (Odds Ratio = 1.43 [95% CI: 1.18; 1.74]). No other variable included in the model was statistically significant.

**Table 5:** Multiple regression model for HIGH-A1c in T1D patients.

	**Regression coefficients**				**Model fit**		**Δ Model fit**
	***b* (SE)**	**95% CI for Odds Ratio**			**χ^2^ (df); *p***	**Nagelkerke *R^2^***	**χ^2^ (df); *p***
		**Lower**	**Odds Ratio**	**Upper**			
**Step 1 (sociodemographic predictors and diabetes specific variables)**					**15.643 (2);** ***p*** **<** **0.001**	**0.206**	
Intercept	0.08(1.29)						
Age	−0.07(0.09)	0.78	0.94	1.12			
Duration of illness	0.26(0.08)*	1.12	1.30	1.50			
**Step 2 (Personality associated variables)**					**37.027 (9);** ***p*** **<** **0.001**	**0.438**	**21.384 (7);** ***p*** **=** **0.003**
Intercept	−1.62(2.68)						
Age	−0.10(0.12)	0.72	0.91	1.14			
Duration of illness	0.35(0.10)^*^	1.18	1.42	1.72			
Depression score (CDI)	0.10(0.74)	0.95	1.10	1.27			
Self-directedness (J-TCI)	−0.01(0.04)	0.92	1.00	1.08			
Body dissatisfaction (EDI)	−0.07(0.04)^†^	0.86	0.93	1.01			
Ineffectiveness (EDI)	0.09(0.14)	0.83	1.09	1.44			
Interoceptive awareness (EDI)	0.30(0.17)^†^	0.97	1.35	1.87			
Asceticism (EDI)	0.31(0.20)	0.92	1.36	2.02			
Blaming others (KIDCOPE) (Ref. not used)	0.45(0.63)	0.46	1.56	5.35			
**Step 3 (Family relation)**					**40.145 (11);** ***p*** **<** **0.001**	**0.468**	**3.118 (2);** ***p*** **=** **0.210**
Intercept	−1.24(2.87)						
Age	−0.12(0.12)	0.70	0.89	1.13			
Duration of illness	0.36(0.10)^*^	1.18	1.43	1.74			
Depression score (CDI)	0.12(0.08)	0.97	1.13	1.32			
Self-directedness (J-TCI)	−0.03(0.05)	0.89	0.98	1.07			
Body dissatisfaction (EDI)	−0.06(0.04)	0.86	0.94	1.02			
Ineffectiveness (EDI)	0.02(0.14)	0.77	1.02	1.34			
Interoceptive awareness (EDI)	0.27(0.17)	0.94	1.30	1.81			
Asceticism (EDI)	0.33(0.20)	0.94	1.40	2.08			
Blaming others (KIDCOPE) (Ref. not used)	0.12(0.03)	0.30	1.13	4.22			
Individual autonomy (SFB)	0.06(0.04)^†^	0.99	1.06	1.14			
Emotional connectedness (SFB)	−0.04(0.03)	0.91	0.96	1.02			

† *p <0.10*,

* *p <0.001; All independent variables of univariate regression models (Supplementary Table S1) with p <0.200 were entered to this multiple logistic regression model. Lines in bold represent statistically significant group differences*.

## Discussion

This is the first study exploring the interplay of depression, personality factors, coping strategies, family relations and quality of life with glycemic control in adolescents with T1D using HbA1c as an objective indicator of illness functioning in a larger sample compared to previous studies.

First, with 41% of patients in the LOW-A1c group, Austria ranks above average in Europe and worldwide with regards to HbA1c levels in young diabetes patients. This is likely due to an excellent health care coverage and subsequently easier access to diabetes services and education in Austria compared to other high-income countries (44, 45).

In the group with HIGH-A1c we found younger age at diabetes onset, longer diabetes duration, and more frequent episodes of severe diabetic ketoacidosis. These results correspond with the results of van Esdonk et al. (46) who found that patients with longer T1D duration were at risk of having higher HbA1c levels. The daily demands of diabetes self-care, including frequent daily blood sugar measurement, multiple injections/boluses, monitoring carbohydrates and exercise to adjust insulin dose have been described as a 24/7 job. To stay alive people with T1D require tracking, monitoring and calculating which can lead to diabetes burnout, neglect and destructive self-care behaviors (47). However, our findings are in contrast with another study showing that later age of onset is a risk factor for higher HbA1c (48).

Second, depression scores were higher in patients with HIGH-A1c compared to patients with LOW-A1c and healthy controls without a chronic condition. The linkage between metabolic control and diabetes duration and depression has been reported in previous studies (24, 49–51). Especially, depression has been associated with elevated HbA1c, higher BMI and episodes of diabetic ketoacidosis (24). This finding seems to be associated with a lower ability of depressed patients to adhere to treatment requirements and with self-destructive or suicidal behaviors like omitting or overdosing insulin (23). Further, an association to elevated blood glucose levels might be due to shared underlying neurobiological factors: hyperactivity of the hypothalamic-pituitary-adrenal (HPA) axis has been reported in both depression and T1D, with elevated stress levels directly affecting glucose metabolism via stress hormones such as glucocorticoids and catecholamines (52). Girls especially are affected with depression, reflecting the gender aspect that girls are more prone to develop internalizing psychiatric disorders which is associated with poor treatment adherence and less frequent blood glucose monitoring (17).

Third, we found lower responsibility, lower ability to set goals and self-acceptance—subsumed as self-directedness—as well as higher ineffectiveness in patients with HIGH-A1c. Self-acceptance, self-esteem and effectiveness represent similar psychological constructs—a positive self-attitude, acknowledging positive and negative aspects of oneself, and not being too self-critical or confused about one's identity (53). Self-esteem and effectiveness may influence responsibility and the ability to set and follow goals in one's life. In other words, people are capable of taking over responsibility for their behavior and actions as well as setting and fulfilling intrinsic motivated goals. Transferring these concepts to adolescents with diabetes, means, accepting oneself, this chronic disease and integrating diabetes in the person's self-identity along with setting goals with regards to specific diabetes management behaviors such as attaining certain HbA1c levels and avoiding hypo- and hyperglycemic episodes (17). Also in previous findings, self-efficacy, self-discipline, conscientiousness and cautiousness have been associated with better diabetes management adherence (26, 54). Other studies found lower self-esteem only in female young adult patients, but not in males (16, 27, 55) and associated with higher Hba1c when patients were in transition from adolescence into adulthood (19). However, the linkage between self-esteem and better adherence to diabetes management has been demonstrated repeatedly, emphasizing the importance of learning to deal with a chronic disease at an early stage of life (56–58). Hence, accepting this chronic disease and integrating it into a positive self-identity seems to be especially important in the period of adolescence, a time frame often associated with insecurity. Lower self-directedness has also been found in adolescent T1D patients with comorbid eating disorders (38)—psychiatric illness connected with low self-esteem known to be associated with deteriorated metabolic control (59, 60).

Furthermore, both harm-avoidance and lower self-directedness are influenced by depression levels (36) and it has been suggested that high harm-avoidance represents an intermediate phenotype for depression but can also be a scarring effect of depression on personality. Also low self-directedness has been considered as a trait marker for depression but can also occur as a consequence of depressive episodes (61).

Fourth, blaming others, classified as negative coping has been applied more often as diabetes related coping strategy in adolescents with HIGH-A1c in our study. This concurs with previous studies showing that in adolescents and young adults, the identification of a passive avoidant coping cluster revealed the most unfavorable profile for metabolic control. Higher levels of avoidance coping style were associated with greater diabetes-related distress leading to fewer blood glucose checks, less frequent self-care behaviors, and poorer glycemic control (62, 63). It has been suggested that emotion-focused coping strategies (such as venting negative emotions) are associated with poorer metabolic control among adolescents with T1D (64). Additionally, influence of favorable and unfavorable coping strategies on HbA1c has been shown previously and seems to also have reversal effects. While higher HbA1C levels and psychological symptoms predicted avoidance coping, active coping prospectively predicted lower HbA1c levels which in turn predicted active coping (63).

Fifth, we found that disadvantageous developmental conditions, lower family cohesions and lower emotional connectedness in families were associated with deteriorated metabolic control, which might be explained by lower emotional attachment in the relationship especially between patients and fathers. Optimal family climate and higher levels of family cohesions have been related to better adherence and glycemic control in adolescents and in young adults (19, 65). The benefits of parental—especially the father's—acceptance and care for a child with T1D and its diabetes management has been suggested previously. When fathers are involved in the care for chronically ill children, treatment adherence of adolescents seems to be better (65). The parent-child relationship during adolescence undergoes significant changes as individuals try to balance autonomy and connectedness. Parents and children who have developed a warm and trusting relationship in which the parent has operated as an effective secure base are at an advantage for developing effective partnerships during adolescence, whereas those with insecure attachments are at greater risk during adolescence (66). The importance of parental support in chronic conditions have been highlighted in other surveys. Research on the contribution of parents to the quality of their child's diabetes self-treatment has found that parents' emotional support of the adolescent, acceptance of the disease, open communication, effective monitoring, and proper conflict resolution are likely to encourage the adolescent's adherence to the treatment regime and thus achieve a better metabolic control (25). Especially, a combined maternal and paternal support seems to be important. Paternal support has been regarded as especially relevant for adolescents with a chronic condition, as fathers tend to encourage independence more than mothers do (25). Our findings add to this developmental perspective and show also that emotional connectedness between father and adolescent is relevant for health outcome as it is associated with lower HbA1c.

Sixth, the results in our survey on the relation between LOW-A1c and better QoL confirm findings of previous studies (32, 67). We found lower overall QoL and deteriorated physical and mental health in patients with HIGH-A1c. Conversely, adolescents with LOW-A1c did not differ from healthy adolescents, suggesting that keeping optimal blood glucose levels may prevent adolescents with T1D from a deterioration in QoL. Poorer QoL predicted subsequent suboptimal glycemic control via less frequent blood glucose monitoring in a prospective study indicating mutual effects (17). Disease-associated and therapy-associated burden was higher in the group with HIGH-A1c levels. However, no differences were found with respect to QoL at school, leisure activities and interests. These finding underline the importance of distinguishing different aspects of QoL and complements the literature showing lower diabetes health related QoL in young women (67, 68). Moreover, other surveys found better quality of life and a higher level of treatment satisfaction in patients treated with insulin pump therapy compared to multiple day injection therapy (32, 69).

Finally, in our multivariate regression model for metabolic control, diabetes duration was associated with HbA1c. To our knowledge, this is the first time all psycho-social factors that potentially play a role for metabolic control are considered altogether. Although no other individual factor was significant, considering personality characteristics and coping strategies in the model helped to differentiate between patients with HIGH-A1c and LOW-A1c.

This result might indicate that female adolescents who have had to manage their chronic disease for a longer period of time might be at risk to fall into diabetes “burn out,” especially in adolescence-a critical period of time where a lot of developmental tasks have to be challenged (body changes, gender identity, establishment of new friendships/partnerships, career decisions for later professions and establishment of core values)—according to Havighurst (70). Univariate analyses indicate that depressive symptoms, low self-evaluation, low self-directedness and the application of negative coping strategies are negatively associated with metabolic control. It is supposed that these factors are interrelated and influence each other. Low self-directedness means the lack of taking responsibility, lack of defining and pursuing aims and lack of self-evaluation. This might be connected to depression and negative coping and fits perfectly into the concept of diabetes burnout characterized by a state of exhaustion and frustration from the daily demands of diabetes self-care which may lead to destructive and neglecting self-care behaviors. The burden of diabetes self-care, lack of achievement in diabetes control and the co-occurrence of critical life stages together with a lack of support system might lead to exhaustion, powerlessness and feelings of detachment (47).

### Limitations

The generalization of our study is limited to female adolescents with T1D, although male adolescents are affected with low HbA1c levels. Higher sample sizes are needed to detect more of the relevant personality factors for high HbA1c with higher precision. Effects of parental autonomy and emotional connectedness have only been examined on an exploratory level; further studies are needed to confirm these findings. Another limitation is the cross-sectional design of the study and more longitudinal studies are needed to address the causal effects of clinical, psychological and familial parameters on metabolic control.

## Conclusion

Female adolescents with early T1D onset, and therefore long diabetes duration who show depressive features, low self-esteem, disadvantageous coping strategies and lower emotional connectedness within the family, especially to the fathers, seem to be particular at risk of having higher HbA1c levels. In order to counteract these adverse mechanisms, it is essential first to routinely assess psychosocial functioning and family conflict and cohesion., psychotherapeutic tools influencing depressive symptoms, self-evaluation, coping and family bonds should be offered to patients and their families. Psychological interventions need to focus on self-acceptance and efficacy in order to promote better glycemic control and should include behavioral components (27, 71). Guiding adolescents to incorporate T1D into their identities may improve self-management as well as mental health (53). Including the parents, especially fathers, in therapeutic interventions is state of the art in child and adolescents' psychiatry and might be favorable for patients with chronic illnesses too. Promoting individual autonomy as well as emotional connectedness between adolescents with T1D and their parents—especially their fathers—contribute essentially to diabetes management efforts in order to not only decrease HbA1c levels but also improve QoL.

## Data Availability Statement

The data that support the findings of this study are available from the corresponding author, Gabriele Berger, upon reasonable request.

## Ethics Statement

The studies involving human participants were reviewed and approved by Ethics Committee of the Medical University of Vienna. Written informed consent to participate in this study was provided by the participants' legal guardian/next of kin.

## Author Contributions

GW and GB performed the research. AK and BR-M designed the study. MZ analyzed the data. GW, AS, and GB wrote the manuscript. All authors have read and approved the final version of the manuscript.

## Funding

This study was supported by a grant from the Jubilaeumsfonds of the National Bank of Austria (Grant number: 11086) given to AK.

## Conflict of Interest

The authors declare that the research was conducted in the absence of any commercial or financial relationships that could be construed as a potential conflict of interest.

## Publisher's Note

All claims expressed in this article are solely those of the authors and do not necessarily represent those of their affiliated organizations, or those of the publisher, the editors and the reviewers. Any product that may be evaluated in this article, or claim that may be made by its manufacturer, is not guaranteed or endorsed by the publisher.
