# Supplementary material for: Personality, Coping and Developmental Conditions in Female Adolescents and Young Adults with Type 1 Diabetes: Influence on Metabolic Control and Quality of Life
Source: Front Psychiatry. 2022 Mar 10;12:809015. doi: 10.3389/fpsyt.2021.809015 (PMC8960069; doi:10.3389/fpsyt.2021.809015)
Supplement: Supplementary file 1 [file Table_1.DOCX]

**Table S1. Univariate logistic regression analyses to predict HIGH-A1c in diabetes patients**

|  | *b* (SE), *p* | 95% CI for Odds Ratio | | |
| --- | --- | --- | --- | --- |
|  | (slopes) | Lower | Odds Ratio | Upper |
| **Sociodemographic variables** | | | | |
| Age | -0.10 (0.06), *p* = .129 | 0.97 | 1.10 | 1.25 |
| Duration of illness | 0.17 (0.06), *p* = .002 | 1.07 | 1.19 | 1.32 |
| Maternal educational (Ref. university, college)  upper secondary education  max. lower secondary education | -0.18 (0.58), *p* = .751  -0.12 (0.58), *p* = .832 | 0.27  0.28 | 0.83  0.88 | 2.57  2.76 |
| Single mother (Ref. in relationship) | -0.15 (0.54), *p* = .787 | 0.30 | 0.86 | 2.50 |
| **Personality associated variables** | | | | |
| Depression score (CDI) | 0.14 (0.04), *p* < .001 | 1.06 | 1.15 | 1.24 |
| Harm-avoidance (J-TCI) | 0.01 (0.02), *p* = .535 | 0.98 | 1.01 | 1.05 |
| Self-directedness (J-TCI) | -0.06 (0.02), *p* = .003 | 0.92 | 0.94 | 0.98 |
| Drive for thinness (EDI) | -0.01(0.04), *p* = .790 | 0.92 | 0.99 | 1.07 |
| Bulimia (EDI) | 0.00 (0.12), *p* = .985 | 0.79 | 1.00 | 1.26 |
| Body Dissatisfaction (EDI) | 0.05(0.03), *p* = .073 | 1.00 | 1.05 | 1.10 |
| Ineffectiveness (EDI) | 0.13(0.06), *p* = .035 | 1.01 | 1.14 | 1.29 |
| Perfectionism (EDI) | 0.03(0.06), *p* = .583 | 0.92 | 1.03 | 1.12 |
| Interpersonal distrust (EDI) | 0.03(0.06), *p* = .584 | 0.92 | 1.03 | 1.16 |
| Interoceptive awareness (EDI) | 0.11(0.07), *p* = .141 | 0.97 | 1.11 | 1.28 |
| Maturity fears (EDI) | 0.02(0.05), *p* = .613 | 0.93 | 1.03 | 1.13 |
| Asceticism (EDI) | 0.22(0.11), *p* = .051 | 1.00 | 1.24 | 1.54 |
| Impulse regulation (EDI) | 0.08(0.07), *p* = .252 | 0.95 | 1.08 | 1.23 |
| Self-blame (KIDCOPE) (Ref. not used) | 0.23 (0.37), *p* = .531 | 0.61 | 1.26 | 2.58 |
| Blaming others (KIDCOPE) (Ref. not used) | 0.81 (0.39), *p* = .037 | 1.05 | 2.25 | 4.83 |
| Wishful thinking (KIDCOPE) (Ref. not used) | -0.02(0.58), *p* = .970 | 0.39 | 0.98 | 2.50 |
| **Family relation** | | | | |
| Individual autonomy (SFIT) | -0.03 (0.02), *p* = .144 | 0.94 | 0.98 | 1.01 |
| Emotional connectedness (SFIT) | -0.04 (0.01), *p* = .011 | 0.94 | 0.96 | 0.99 |

Note: CDI = Child Depression Inventory, J-TCI = Junior Temperament and Character Inventory, EDI-2 = Eating Disorder Inventory-2, KIDCOPE = Quality of Life Questionnaire, SFIT = Subjective Family Image Test
All predictors of univariate regression models with *p* < .200 were entered to the multiple logistic regression model
