# Supplementary material for: Personality, Coping and Developmental Conditions in Female Adolescents and Young Adults with Type 1 Diabetes: Influence on Metabolic Control and Quality of Life
Source: Front Psychiatry. 2022 Mar 10;12:809015. doi: 10.3389/fpsyt.2021.809015 (PMC8960069; doi:10.3389/fpsyt.2021.809015)
Supplement: Supplementary file 2 [file Table_2.DOCX]

**Table S2. Multiple regression model to predict HIGH-A1c in diabetes patients**

|  | **Regression coefficients** | | | | |  | **Model fit** | |  | **Δ Model fit** |
| --- | --- | --- | --- | --- | --- | --- | --- | --- | --- | --- |
|  | *b* (SE) | 95% CI for Odds Ratio | | | |  | χ²(df); *p* | Nagelkerke *R²* |  | χ²(df); *p* |
|  |  | Lower | Odds Ratio | | Upper |  |  |  |  |  |
| **Step 1 (sociodemographic predictors and diabetes specific variables)** | | | | | |  | **15.643(2); *p* < .001** | **.206** |  |  |
| Intercept | 0.08(1.29) |  | |  |  |  |  |  |  |  |
| Age | -0.07(0.09) | 0.78 | | 0.94 | 1.12 |  |  |  |  |  |
| Duration of illness | 0.26(0.08)*** | 1.12 | | 1.30 | 1.50 |  |  |  |  |  |
| **Step 2 (Personality associated variables)** | | | | | |  | **37.027(9); *p* < .001** | **.438** |  | **21.384(7); *p* = .003** |
| Intercept | -1.62(2.68) |  | |  |  |  |  |  |  |  |
| Age | -0.10(0.12) | 0.72 | | 0.91 | 1.14 |  |  |  |  |  |
| Duration of illness | 0.35(0.10)*** | 1.18 | | 1.42 | 1.72 |  |  |  |  |  |
| Depression score (CDI) | 0.10(0.74) | 0.95 | | 1.10 | 1.27 |  |  |  |  |  |
| Self-directedness (J-TCI) | -0.01(0.04) | 0.92 | | 1.00 | 1.08 |  |  |  |  |  |
| Body Dissatisfaction (EDI) | -0.07(0.04)^✝^ | 0.86 | | 0.93 | 1.01 |  |  |  |  |  |
| Ineffectiveness (EDI) | 0.09(0.14) | 0.83 | | 1.09 | 1.44 |  |  |  |  |  |
| Interoceptive awareness (EDI) | 0.30(0.17)^✝^ | 0.97 | | 1.35 | 1.87 |  |  |  |  |  |
| Asceticism (EDI) | 0.31(0.20) | 0.92 | | 1.36 | 2.02 |  |  |  |  |  |
| Blaming others (KIDCOPE) (Ref. not used) | 0.45(0.63) | 0.46 | | 1.56 | 5.35 |  |  |  |  |  |
| **Step 3 (Family relation)** | | | | | |  | **40.145(11); *p* < .001** | **.468** |  | **3.118(2); *p* = .210** |
| Intercept | -1.24(2.87) |  | |  |  |  |  |  |  |  |
| Age | -0.12(0.12) | 0.70 | | 0.89 | 1.13 |  |  |  |  |  |
| Duration of illness | 0.36(0.10)*** | 1.18 | | 1.43 | 1.74 |  |  |  |  |  |
| Depression score (CDI) | 0.12(0.08) | 0.97 | | 1.13 | 1.32 |  |  |  |  |  |
| Self-directedness (J-TCI) | -0.03(0.05) | 0.89 | | 0.98 | 1.07 |  |  |  |  |  |
| Body Dissatisfaction (EDI) | -0.06(0.04) | 0.86 | | 0.94 | 1.02 |  |  |  |  |  |
| Ineffectiveness (EDI) | 0.02(0.14) | 0.77 | | 1.02 | 1.34 |  |  |  |  |  |
| Interoceptive awareness (EDI) | 0.27(0.17) | 0.94 | | 1.30 | 1.81 |  |  |  |  |  |
| Asceticism (EDI) | 0.33(0.20) | 0.94 | | 1.40 | 2.08 |  |  |  |  |  |
| Blaming others (KIDCOPE) (Ref. not used) | 0.12(0.03) | 0.30 | | 1.13 | 4.22 |  |  |  |  |  |
| Individual autonomy (SFB) | 0.06(0.04)^✝^ | 0.99 | | 1.06 | 1.14 |  |  |  |  |  |
| Emotional connectedness (SFB) | -0.04(0.03) | 0.91 | | 0.96 | 1.02 |  |  |  |  |  |

^✝^ *p* < .10, * *p* < .05, ** *p* < .01, * *p* < .001; All predictors of univariate regression models (Table S1) with *p* < .200 were entered to this multiple logistic regression model
